# *TP53* Gene Status Affects Survival in Advanced Mycosis Fungoides

**DOI:** 10.3389/fmed.2016.00051

**Published:** 2016-11-11

**Authors:** Gitte Wooler, Linea Melchior, Elisabeth Ralfkiaer, Lise Mette Rahbek Gjerdrum, Robert Gniadecki

**Affiliations:** ^1^Department of Pathology, Zealand University Hospital, Roskilde, Denmark; ^2^Department of Pathology, Rigshospitalet, Copenhagen, Denmark; ^3^Department of Dermatology, Bispebjerg Hospital, Copenhagen, Denmark; ^4^Division of Dermatology, Faculty of Medicine and Dentistry, University of Alberta, Edmonton, AB, Canada

**Keywords:** p53 mutation, mycosis fungoides, survival rate, sequencing data analysis, cutaneous lymphoma

## Abstract

*TP53* is frequently mutated in different types of neoplasms including leukemia and lymphomas. Mutations of *TP53* have also been reported in mycosis fungoides (MF), the most common type of cutaneous lymphoma. However, little is known about the frequency, spectrum of mutations, and their prognostic significance in MF. In this study, we have optimized the protocol for Sanger sequencing of *TP53* using DNA extracted from archival paraffin-embedded biopsies. Of 19 samples from patients with stage IIB MF or higher, 31% harbored mutations in *TP53*. Overall survival of the patients with mutated *TP53* was significantly shorter than median survival in the age- and stage-matched patients treated in our Institution. Distribution of mutations was heterogenous in *TP53* exons; however, C > T transitions were common suggesting the causal role of ultraviolet radiation. We propose that *TP53* mutation status would be useful for risk stratification of patients with advanced MF.

## Introduction

Mycosis fungoides (MF) is the most common form of cutaneous T-cell lymphoma (CTCL) and in most cases runs an indolent course. However, approximately 20% of patients would progress to a widespread disease with multiple skin tumors and extracutaneous involvement with dire outcome (1). Male gender, young age, and folliculotropic subtype are risk factors for progression, but their predictive value is low (1, 2). There are currently no robust biomarkers that predict the course of the disease.

The tumor suppressor gene *TP53* is central in tumorigenesis and regarded as a master regulator of several signaling pathways involved in this process. *TP53* is mutated in more than 50–70% of all solid tumors and in approximately 10–20% in hematological malignancies (3). While low-grade lymphoid neoplasms reveal low p53 mutation rates, lymphomas and leukemias (in particular chronic lymphatic leukemia) with an aggressive clinical course demonstrate higher frequencies (4–7). To this end, a strong correlation was found between p53 functional status and clinical outcomes in lymphoma, such as mortality or resistance to chemotherapy (8, 9). *TP53* may be mutated in a proportion of patients with another type of CTCL, Sezary syndrome, but mutations are not predictive for the course of the disease (10). Less is known about *TP53* gene status in MF. Previous studies suggested that p53 overexpression and mutation is uncommon in early MF (11–13) but can be found in advanced stages indicating prognostic significance (14, 15). It has also been suggested that p53 mutations in MF are caused by ultraviolet radiation (11, 14, 16). Therefore, in this study we wished to elucidate the frequency and potential role of *TP53* mutations in MF according to disease stage and course. We have developed a method, which enables *TP53* sequencing from paraffin-embedded samples from a relatively large number of patient samples.

## Materials and Methods

### Materials

In this retrospective study, we reviewed clinical data and histopathology of 157 patients diagnosed with MF in Denmark from 1980 to 2013 and registered in the Danish pathology registry database (Patobank) and The Danish Cutaneous Lymphoma Database.^1^ The diagnosis was made by correlation between clinical findings and histological examination of skin biopsies (17). Staging was performed according to ISCL/EORTC criteria (18). Also, 30 patients had MF stage IIB or higher, and tissue for p53 analysis was available in 19 cases (Table 1). Control materials included CTCL cell lines SeAx, MyLa200, and MF1885, together with biopsies from solid tumors with known p53 mutations (19).

**Table 1:** **Patients with MF included in mutation analysis of *TP53***.

No.	Gender/age (years)	Clinical stage	Mutation status of *TP53*	Disease duration at highest stage
1	Male/67	T3N3M1B0	Mutated: exon 4: c.100C > T (p.P34S); exon 4: c.254C > T (p.P85L); exon 4: c.430C > T (p.Q144stop)	7 years, DOD
2	Female/91	T3N0M0B0	Mutated: exon 8: c.811G > A (p.E271K); exon 5 and 6 unable to amplify	1 year, DOD
3	Female/67	T4N3M0B2	No mutations	10 years, DOD
4	Male/72	T3N2M0B0	No mutations	2 years, AWD
5	Female/89	T3N0M0B0	No mutations	8 years, DOC
6	Female/56	T4N1M0B1	No mutations	5 years, DOD
7	Male/69	T3N3M1B0	No mutations: exon 5 and 6 unable to amplify	3 years, DOD
8	Male/76	T4N3M0B1a	No mutations	3 years, DOD
9	Female/65	T3N1M0B0	No mutations	14 years, AWD
10	Male/83	T3N0M0B0	No mutations	11 years, AWD
11	Female/55	T3N0M0B0	No mutations	5 years, AWD
12	Male/75	T3N0M0B0	No mutations in exon 7, 10: exon 4, 5, 6, 8, and 9 unable to amplify	3 years, DOC
13	Male/64	T4N3M0B2	Mutated: exon 5: c.449C > T (p.T150I)	4 years, DOD
14	Female/74	T3N0M0B0	No mutations	6 years, AWD
15	Female/71	T3N0M0B0	No mutations	12 years, AWD
16	Female/63	T3N3M0B0	No mutations: exon 4, 5, and 6 unable to amplify	5 years, DOC
17	Male/76	T3N0M1B0	Mutated: exon 5: c. 502C > T (p.H168Y); exon 5: c. 530C > A (p.P178H); exon 6: c. 670G > T (p.E224Stop)	3 years, DOD
18	Male/99	T3N0M0B0	Mutated: exon 8: c.818G > A (p.R273H)	2 years, DOC
19	Male/66	T3N2M0B0	Mutated: exon 5: c.457C > T (p.P153S); exon 5: c.461G > A (p.G154D)	5 years, DOD

*AWD, alive with disease; DOD, dead of disease; DOC, dead of other causes*.

### TP53 Sequencing

Genomic DNA was extracted from 4 μm × 5 μm paraffin-embedded biopsies from lesional skin and purified using the DNA Sample Preparation Kit (Roche Life Science, Basel, Switzerland) according to the manufacturer’s instructions. Concentration and purity was measured on a Nanodrop 2000 instrument. PCR amplification of *TP53* exon 4–10 is performed in five separate nested PCR reactions according to Table 2. The primers are designed using Primer 3 software (20, 21) and purchased from MWG Eurofins.^2^ The nest 1 reactions contained, in a volume of 15 μl, 0.33 μmol/l of each nest 1 primer, 7.5 μl RedEx PCR master mix (Sigma-Aldrich, St Louis, MO, USA) and 50–100 ng genomic DNA and was amplified using the following PCR conditions: initial denaturating at 95°C for 5 min, 25 cycles at 95°C for 30 s, 55°C for 30 s, and 72°C for 30 s, and a final extension at 72°C for 10 min. The nest 2 reactions contained, in a volume of 50 μl, 0.4 μM of each nest 2 primer, 25 μl RedEx PCR master mix (Sigma-Aldrich) and 1 μl 100-fold diluted nest 1 product. The nest 2 PCR conditions were the same as for the nest 1 reactions except that a total of 35 cycles were used. All five nest 1 and nest 2 reactions uses the same amplification settings. The PCR products were purified using the QIAquick PCR purification kit (Qiagen, Hilden, Germany). Sequence reactions were carried out using T3 and T7 primers, 25 ng purified PCR products, and BigDye Terminator 3.1 Cycle Sequencing Kit chemistry (Life Technologies, Carlsbad, CA, USA) for BigDye incorporation and were subsequently sequenced on an ABI3500DX DNA sequenator according to the manufacturer’s instructions.

**Table 2:** **Primer sequences for sequencing of *TP53* exon 4–10**.

Exon		Primer sequence	Fragment (bp)
4	F-N1	5′-CCATGGGACTGACTTTCTGC	534
	R-N1	5′-GAGGAATCCCAAAGTTCCAA	
	F-N2	5′-CTGGTAAGGACAAGGGTTGG	457
	R-N2	5′-AGAAATGCAGGGGGATACG	
5 + 6	F-N1	5′-GGAGGTGCTTACGCATGTTT	588
	R-N1	5′-GGGAGGTCAAATAAGCAGCA	
	F-N2	5′-GCCGTCTTCCAGTTGCTTTA	506
	R-N2	5′-GCCACTGACAACCACCCTTA	
7	F-N1	5′-CCTGCTTGCCACAGGTCT	294
	R-N1	5′-TGATGAGAGGTGGATGGGTAG	
	F-N2	5′-TGCTTGCCACAGGTCTCC	236
	R-N2	5′-GGTCAGAGGCAAGCAGAGG	
8 + 9	F-N1	5′-GGGAGTAGATGGAGCCTGGT	486
	R-N1	5′-CCCCAATTGCAGGTAAAACA	
	F-N2	5′-GGGACAGGTAGGACCTGATTT	431
	R-N2	5′-AAGAAAACGGCATTTTGAGTG	
10	F-N1	5′-TGCATGTTGCTTTTGTACCG	300
	R-N1	5′-GAAGGCAGGATGAGAATGGA	
	F-N2	5′-TGCATGTTGCTTTTGTACCG	263
	R-N2	5′-CCTAGGAAGGCAGGGGAGTA	

*The nest 2 primers contain T3 and T7 tags on F-N2 and R-N2, respectively*.

### Statistical Analysis

The overall survival (time in years from the diagnosis to death of any cause or to the last observation) was calculated for all 30 patients with stage >IIB MF. Cox logistic regression was performed using SPSS statistical package (version 22, IBM Corporation, Armonk, North Castle, NY, USA) using enter, forward, or backward methods. Kaplan–Meier plot was generated in SPSS to compare survival curves between patients with mutated and germline *P53. p* ≤ 0.05 was considered significant.

## Results

In 6 out of 19 cases (31%), 1 or several mutations could be identified, exhibiting a heterogeneous pattern with mutations in different exons at various codon sites (Table 1).

**Figure 1:**
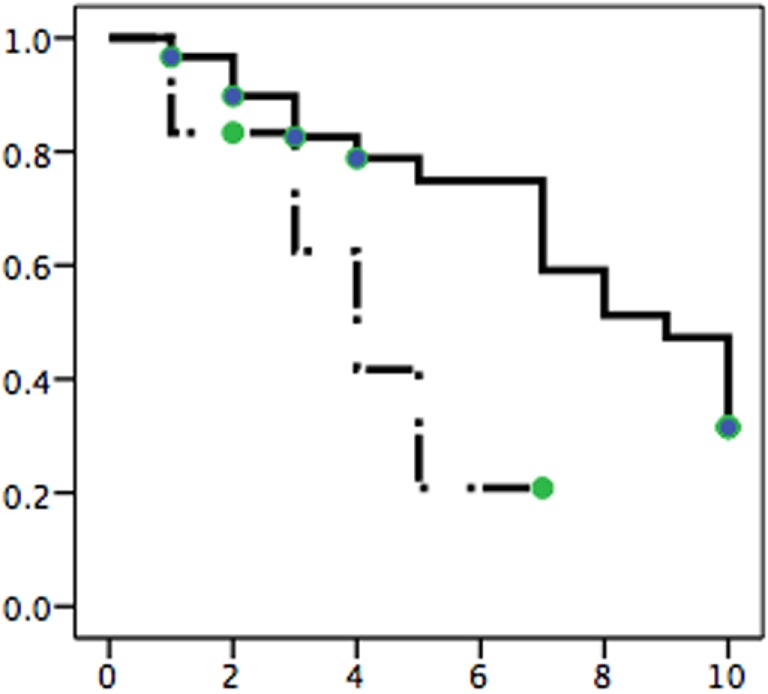
**Comparison of survival in MF patients with normal *TP53* (solid line) and mutated gene (broken line)**. Six patients with mutated *TP53* (Table 1) are compared against the control group of 30 patients with stage IIB MF or higher. The difference in survival is significant (*p* = 0.05) after adjustment for stage, age, and gender by Cox logistic regression. *x*-axis – years, *y*-axis – probability of survival.

To investigate whether *TP53* mutations had impact on survival, we compared patients with mutated and normal *TP53* by Kaplan–Meier analysis. Because the sample size was small, we decided to compare survival of the 6 patients with mutated p53 to all 30 patients with stage >IIB MF treated in our center in the same time frame (14 women, 16 men; mean age 71.8 years, SD 12.6). As shown in Figure 1, patients with mutated *TP53* had a shorter survival than the controls. The statistically significant difference (*p* = 0.05) was maintained when data were adjusted to age, stage, and gender by Cox logistic regression. Adjusted odds ratio for death in the presence of mutated *P53* was 2.99 (95% confidence interval 1.12–8.00, Cox regression) comparing to the control group.

## Discussion

The protocol developed in this study allowed for PCR amplification and Sanger sequencing of *TP53* in archival formalin-fixed and paraffin-embedded samples of MF. Only in 4 out of 19 samples, we were unable to amplify one or several exons, which is most probably due to sample age and DNA crosslinking.

The frequency of mutations in stage IIB or higher was 31%, which is similar to what has been reported previously (11, 16). In contrast, no mutations have been identified in a recent study of McGirt et al. (14) who reported Pro72Arg polymorphism to be more common in MF. Mutation analysis did not reveal any distinctive pattern of alterations in *TP53*. None of the earlier reported mutations were identical to our findings, and our cases harbored only one of the known hotspot mutations (R273H in case 18). In a recent study, Choi et al. reported 7 different mutations located at different sites in 7 out of 40 analyzed patients, including the well-known R273H hotspot (22). Our cases revealed mainly missense single nucleotide mutations resulting in stop codons (cases 1 and 17). Frequent C > T transitions seem to support the hypothesis that *TP53* mutations demonstrate an UV B signature, which might be related to the effect of phototherapy given repeatedly in the earlier stages of the disease (11, 14, 16).

*TP53* mutations seem to be associated with worse outcome (higher overall mortality). However, this analysis should be interpreted with caution since our sample is small, and the presence of mutations may reflect more intensive use of phototherapy in patients with aggressive, difficult to control disease. Larger sample and careful matching with the control patients with normal *TP53* status will be required to elucidate this issue.

## Author Contributions

Design of the study: RG, LG, and LM; data collection: LM, ER, LG, and RG; analysis of data and full access to raw data: RG, LG, and LM; drafting of the manuscript: GW, LM, LG, and RG; and revision of manuscript for important intellectual content: RG, LG, LM, GW, and ER.

## Conflict of Interest Statement

The authors declare that the research was conducted in the absence of any commercial or financial relationships that could be construed as a potential conflict of interest. The reviewer [UK] and handling Editor declared their shared affiliation, and the handling Editor states that the process nevertheless met the standards of a fair and objective review.

## Footnotes

^1^www.cldbase.com.

^2^www.eurofinsgenomics.eu.
